# Vaccine advertising: preach to the converted or to the unaware?

**DOI:** 10.1038/s41746-021-00395-7

**Published:** 2021-02-11

**Authors:** Masha Krupenkin, Elad Yom-Tov, David Rothschild

**Affiliations:** 1 Boston College, Boston, MA USA; 2 Microsoft Research, Herzeliya, Israel; 3 Microsoft Research, New York, NY USA

**Keywords:** Information technology, Health care

## Abstract

Encouraging people to vaccinate is a challenging endeavor, but one which has tremendous public health benefits. Doing so requires overcoming barriers of awareness, availability, and (sometimes) vaccine hesitancy. Here we focus on nudging people to vaccinate through online advertising. We conducted a pre-registered online ads campaign encouraging people to vaccinate against three diseases: influenza, human papillomavirus, and herpes zoster. Ads were shown to ~69,000 people and were compared to similar ads shown to 8.6 million people. Outcome measures were clicks on ads and future searches for relevant terms. We find that ads have two main effects: First, a congruence effect whereby ads increase the likelihood of clicks and future searches by up to 116% in people who express an interest in the disease or the vaccine. Most commercial vaccine advertising is aimed entirely at this population. Second, we observed a priming effect, where ads shown to people who were searching for terms unrelated to the vaccine could be encouraged to click on them (odds ratios of 7.5–33.0) and, more often, search for the vaccine later (hazard ratios of 6.9–157.3). We provide analysis for optimizing vaccine advertising campaign budgets to balance the two populations. These findings demonstrate that digital advertising campaigns should consider not just advertising to direct keywords or to individuals that look exactly like existing customers, but consider tangential keywords that draw a wider target population who are likely earlier in their conversion funnel, thus increasing the number of people who vaccinate and maximizing vaccines uptake.

## Introduction

Vaccines are likely the most effective public health intervention ever devised, preventing an estimated 2–3 million deaths every year^1^. Nevertheless, vaccination coverage is far from complete: In the USA, only 35% of relevant adults vaccinated against Herpes Zoster (shingles), 52% of females (21% of males) against human papillomavirus (HPV), and 45% of adults were vaccinated against influenza (flu)^2^.

One way to increase vaccination coverage is by advertising these vaccines to increase awareness to them and to inform people of their necessity. Specifically, online advertising offers a highly efficient way to tune adverts to inferred user needs and demographics. For example, in search advertising ads are served based on query keywords which indicate intent^3^. For this reason, spending on search advertising (also known as sponsored ads) in 2019 in the U.S. was valued at US$36.5 billion^4^ and US$109.9 worldwide^5^.

Ads have been used to steer people towards healthier behaviors, including smoking cessation^6^, weight loss^7^, and reduction in harm from eating disorders^8^. Directly measuring the effect of an online advertising campaign is difficult (e.g.,^9^). Thus, more often a proxy for the intended outcome is used. Web searches have been shown to be a powerful predictor of a wide variety of future behaviors. This includes a number of health-related behaviors and metrics, such as prescription drug utilization^10^, usage of electronic cigarettes^11^, mask-wearing^12^, as well as rates of both infectious^13,14^ and non-infectious diseases^15,16^. However, the utility of search as a predictive tool extends far beyond health behaviors. Web search data has also been used to predict home and auto purchasing behavior^17^, airline travel^18^, unemployment^19^, voter registration^20^, and suicide^21^. Given this plethora of predictions, it is reasonable to infer that searches for vaccines should be considered meaningfully related to actual vaccination behavior, in addition to raising awareness to (in our case) a vaccine. The only stronger proxy we are aware of, for vaccine advertising, is the work by Mohanty et al.^22^, which used the appearance of a person’s phone within a vaccination clinic as an indication that the person vaccinated.

Research into online advertising campaigns promoting vaccines focused on messaging which stressed the benefits of immunization or on providing information on opportunities for vaccination (see, for example^22,23^). Little attention has been given to the ways in which users can be reached and on ways to identify user intent. As we show below, most commercial vaccine advertising is aimed at people already expressing awareness of the vaccine, if not outright intention to vaccinate. We hypothesize that the reason for this focus is to nudge people to use specific vaccine types or vaccine providers. In marketing terms, these people are already in the conversation funnel, on their way to buying a vaccine, just debating over which one and where^24^.

This paper thus speaks to a robust literature on where search ads are in the conversion funnel, especially in relation to their brand specificity. The bottom of the funnel normally has very specific, more rare keywords, as people search for specific products they are going to buy, making them ripe to click on ads versus organic (non-paid) links^24^. This also speaks to the research on category searches^25^ which tend to diminish when the brands are well known. With vaccines the brands are not known (with exception of locations for flu shots, but those searches are relatively rare), so the searches are almost always categorical. Thus, in some ways, the vaccines may feel more like indirect sales, than direct sales, benefiting from varied, less direct keywords^26^.

We focus on an extreme example of indirect sales, people at the very top of the funnel who are unaware of a relevant vaccine (or even the disease): this is especially important for a stakeholder that may not care about which vaccine they receive or where, but simply that they obtain it. This is complicated by the fact that a large percentage of the relevant population for vaccination is unaware of the need to vaccinate with many unaware of the medical condition itself.

Therefore, we ask, is it possible to drive people who are unaware of a vaccine towards inoculation, and if so, what should the balance between advertising to people who are unaware, compared to those who are aware but have not yet vaccinated, be so as to maximally raise the number of vaccinated people?

## Results

### Campaign statistics

We ran advertising campaigns for vaccinations against three diseases: influenza, HPV, and herpes zoster.

**Fig. 1:**
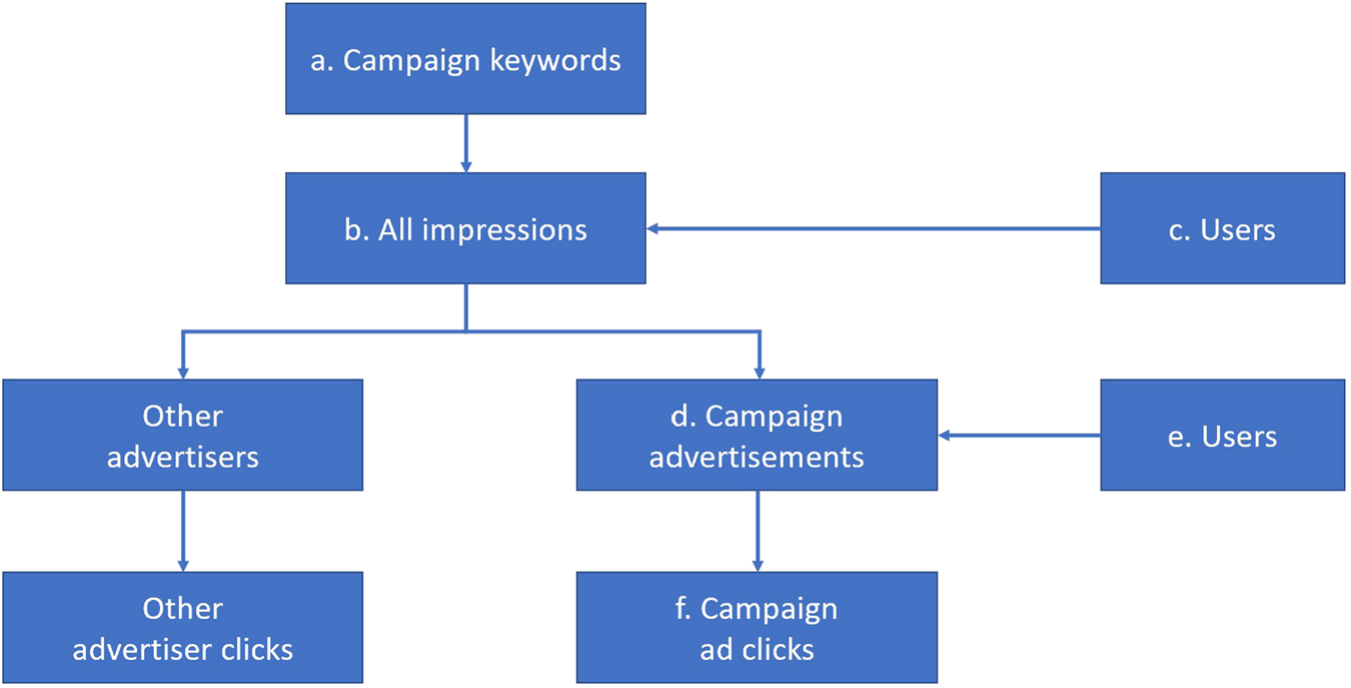
Flowchart of the advertising campaign. Letters refer to the numbers shown in Table 1.

Figure 1 exemplifies an advertising campaign and its execution. As described in the Methods, once campaign keywords are selected (a) the ads are shown to users who query using these keywords. Such campaign showings are called impressions (b). The impressions are divided between those where the campaign ads are shown (d) and where the ads of other advertisers are displayed. Each ad can be clicked by a user (f).

Table 1 shows the statistics of the three campaigns, including the dates of the campaigns, the number of distinct ad texts we created and the number of search terms used to trigger ads, number of times that the ads were shown and clicked, and the number of times that other ads were shown in response to the same queries as those for which the campaign ads were shown, denoted by “all ads”. The statistics in Table 1 refer to the steps in Fig. 1.

**Table 1:** Statistics of the advertising campaign and of concurrently running vaccine-related ads from other advertisers.

Parameter	Influenza	HPV	Herpes Zoster
Dates	8 Oct. – 27 Nov. 2019	13 Jan. – 4 Apr. 2020	13 Jan. – 4 Apr. 2020
Number of campaign ads	30	10	10
Number of campaign keywords^a^	644	474	200
with sufficient impressions	69	27	26
Impressions on			
all vaccine-related ads^b^	19.4M	7.4M	21.4M
campaign ads^d^	71,799	12,804	20,762
Clicks on			
all vaccine-related ads	602,542	187,092	871,077
campaign ads^f^	926	522	947
Number of users exposed to			
all vaccine-related ads^c^	3.4M	1.7M	3.5M
campaign ads^e^	45,715	8845	14,319

Keywords with sufficient impressions refer to keywords which received at least 100 impressions in influenza and herpes zoster campaigns, and at least 25 impressions in the HPV campaign. Superscript letters refer to the steps in the flowchart on Fig. 1.

Among all vaccine-related ads, 98.1% of influenza ads were shown in response to vaccine- or disease-related keywords. Over 99.99% of HPV and Herpes Zoster ads were shown in response to such keywords.

Crowdsourcing coding of terms for their relatedness to the disease or vaccine produces very clear results for many terms being unrelated. On a scale of 1 (for “Definitely NOT searching for [vaccine]”) to 5 (for “Definitely searching for [vaccine]”), 40 of 68 Flu, 14 of 27 HPV, and 19 of 26 Herpes Zoster had means of 1 (i.e., every coder gave them a 1). On the top end there is less precision as coders gave terms for the actual vaccines means between 2.5 and 5.0 with an average of 4.2, and terms for the underlying illnesses between 1.7 and 4.2 with an average of 3.2. Sample terms and their scores are shown in Supplementary Data 1.

### Priming effect

We calculated the term similarity, CTR and the percentage of future searches for the keywords of each campaign. Table 2 shows the correlation between term similarities and the two latter variables. CTR is uncorrelated with term similarity. However, term similarity is strongly correlated with the percentage of future searches. Note that the distribution of term similarities is not uniform (see Supplementary Data 1), which may lead to an underestimation of the actual associations.

**Table 2:** Correlation of term similarity with CTR and with the percentage of future searches for the vaccine.

Parameter	Influenza	HPV	Herpes Zoster
CTR	0.10	−0.03	0.10
Future searches	0.63^*^	0.63^*^	0.88^*^
*n*	44	22	23

*n* the number of terms assessed for each campaign.

*Statistically significant correlations (*P* < 0.05 with Bonferroni correction).

Using a Cox proportional hazard model, we evaluated the likelihood that a searcher who queried for one of the target terms (e.g., the condition searches) would later search for vaccines. Searches for terms which were not in the list of condition searches were marked as non-condition searches. The dependent variable is the time to future search from the original ad impression. Impressions that were not followed by a vaccine search from the same user were counted as censored. Table 3 shows the results of this model. We find that in all three cases, treated searchers were more likely to search for vaccines in the future than untreated searchers, and, most important: treated non-condition searchers were much more likely to search than untreated non-condition searchers.

**Table 3:** Hazard ratio of a Cox hazard model of post-ad vaccine searches. Numbers shown are hazard ratios.

Attribute	Influenza	HPV	Herpes Zoster
Treatment	1.031 [1.008, 1.055]	1.714 [1.646, 1.787]	2.219 [2.168, 2.272]
Orig query non-condition	0.034 [0.034, 0.035]	0.003 [0.003, 0.004]	0.006 [0.006, 0.006]
Treatment × non-condition	6.945 [6.467, 7.459]	157.275 [145.415, 169.984]	30.508 [28.024, 33.194]

Numbers shown are hazard ratios. All results are statistically significant parameters (*P* < 0.05 with Bonferroni correction). Orig query non-condition refers to users whose original search was neither a vaccine nor disease related query. Treatment refers to users who received our ad, as opposed to control users, who received standard Bing ads. Running the regression without the treatment × condition interaction does not substantively change the hazard ratios for treatment or non-condition variables.

Not only was the effect of treatment on post-ad vaccine searches greater, this effect extended to post-ad vaccine clicks. Table 4 shows the results of a logistic regression model for post-ad vaccine clicks. Searchers were coded as a 1 if they did a post-ad vaccine search and clicked an ad or organic (unpaid search) link in the search results, and 0 if they either did not do a post-ad vaccine search or did not click on any of the results. Table 4 presents the results of this model, which show that our ads generated a significant increase in post-ad clicks on vaccine-related links.

**Table 4:** Logistic regression model of future vaccine clicks.

Attribute	Influenza	HPV	Herpes Zoster
Treatment	0.920 [0.881, 0.962]	1.551 [1.409, 1.709]	2.048 [1.965, 2.137]
Orig query non-condition	0.035 [0.034, 0.036]	0.003 [0.003, 0.004]	0.007 [0.006, 0.007]
Treatment × non-condition	7.533 [6.603, 8.595]	43.903 [31.293, 61.566]	33.016 [27.632, 39.474]

Numbers shown are odds ratios. All results are statistically significant parameters (*P* < 0.05 with Bonferroni correction). Standard errors clustered by user. Running the regression without the treatment and condition interaction does not substantively change the hazard ratios for treatment or non-condition variables.

**Fig. 2:**
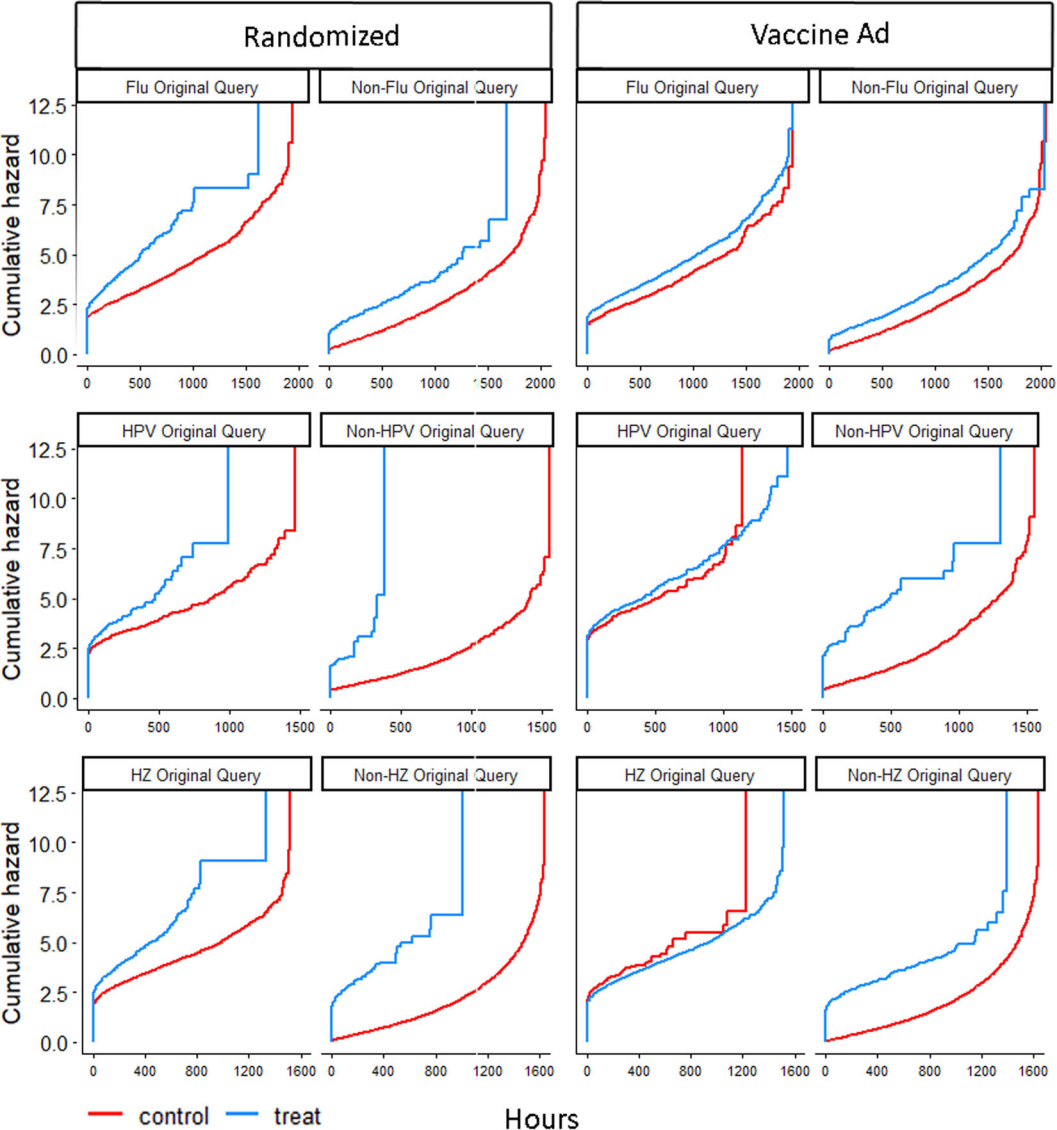
Cumulative hazard curves for post-ad vaccine searches. The randomized column displays results of the ad experiment, with users who saw our ads counted as “treated”. Vaccine Ad column displays results of robustness check, with user who saw any vaccine ad counted as “treated”.

Figure 2 shows the cumulative hazard curves for post-advertisement vaccine searches, with the horizontal axis representing hours elapsed since since the search for a relevant term, stratified by whether the user queried for the disease or vaccine or not, and whether the user saw our campaign ads. As the figures show, treatment always increases the likelihood of future vaccine searches, especially in the near future (see also ref. ^27^). Curves for the control population which queried for the vaccine are significantly higher than for those who didn’t search for the vaccine. However, treatment of the latter population increases their likelihood of future vaccine searches to almost the same level as that of the control population which did search for the vaccine, clearly demonstrating increased awareness in this population.

Note that, in the case of non-condition original query, we see a large difference at time zero between the treated and control units. This is because for the non-condition searches, people are making a search unrelated to the vaccine or disease, and in the control, they are also not shown a vaccine ad. As such, if any of these people do eventually search for vaccines, it is likely to be long after their original search. On the other hand, a significant proportion of the non-condition users who were in the treatment group and received a vaccine ad on their search term did indeed search for vaccines shortly after seeing the ad, providing evidence of a priming effect of the vaccine ad on vaccine searches.

### Congruence effect of ads

We refer to the congruence effect to describe the change in the likelihood of clicks and future searches caused by the display of relevant ads to people who express an interest in the disease or the vaccine. We evaluated this effect by modeling the likelihood that links to ads or, separately, organic (unpaid search) links will be clicked, as a function of their parameters as well as those of the remaining page, taking into account whether additional ads and/or organic links pointed to the same website as that of the considered link (ad or organic).

Specifically, the model parameters account for the placement of the page or ad, as calculated by the rank of the ad or organic link in the page, where rank 1 means that they are placed highest. Model parameters of the page include the total number of ads and organic links on the page and the probability of the query among all queries. Model parameters of congruence include the fraction of ads or links on the search page which refer to the same website as that of the examined link.

The attributes we evaluated were:

- The number of ads displayed on the page
- The number of organic links displayed on the page
- The rank of the ad or the organic link
- Query popularity: Logarithm of the number of times this query text appears in our data
- Fraction of additional ads pointing to the same website
- Fraction of additional organic links pointing to the same website

Table 5 shows logistic regression models for the likelihood of clicks, separately for ads and for organic links. As the table shows, pages and ads which are higher in the page (lower rank) receive more clicks (as also shown in ref. ^28^). Additional ads are correlated with more clicks on ads but fewer organics, whereas fewer organic links are correlated with a higher likelihood of clicks on both ads and organic links.

**Table 5:** Logistic regression model of clicks on ads and organic links for vaccination queries.

Attribute		Influenza		HPV		Herpes Zoster
	Ads	Organic	Ads	Organic	Ads	Organic
Rank	0.44 [0.43–0.46]	0.61 [0.60–0.62]	0.56 [0.55–0.56]	0.65 [0.65–0.66]	0.28 [0.27–0.28]	0.57 [0.56–0.58]
Number of ads	1.13 [1.11–1.14]	0.91 [0.90–0.92]	1.19 [1.19–1.20]	0.93 [0.92–0.93]	1.29 [1.28–1.31]	0.89 [0.88–0.90]
Number of organic	1.01‡ [0.97–1.06]	0.88 [0.88–0.89]	0.91 [0.91–0.92]	0.95 [0.95–0.96]	0.99‡ [0.97–1.00]	0.97 [0.97–0.98]
Query popularity	1.00‡ [0.96-1.05]	0.90 [0.88–0.92]	1.09 [1.08–1.11]	0.87 [0.87–0.88]	1.02‡ [1.01-1.04]	0.90 [0.89–0.91]
Same-site ads	1.61‡ [1.26–2.07]	1.00‡ [0.89–1.13]	0.51 [0.45–0.57]	1.21 [1.15–1.27]	1.42 [1.28–1.58]	3.25 [2.60–4.07]
Same-site organic	0.04‡ [0.00-0.56]	0.83 [0.76–0.90]	3.05 [2.27–4.09]	0.38 [0.36–0.40]	0.35‡ [0.17-0.74]	1.31 [1.21–1.43]

The numbers shown are the odds ratios. Same-site ads/links refer to the number of additional ads/links to the same website divided by the total number of ads/links on the page, pointing to the same website as that of the considered link. All results are statistically significant parameters (*P* < 0.05 with Bonferroni correction) except those marked by ‡.

Interestingly, more ads pointing to the same website have a mixed effect on clicks, in some cases (Herpes Zoster) associated with more clicks on ads and in some fewer (HPV), whereas their appearance is correlated with an increasing likelihood of clicks on organic links. Additional organic links from the same website are associated with more clicks on ads in the case of HPV, and less on the organic links in the case of influenza and HPV, but not Herpes Zoster. Thus, it would appear that for vaccine-related queries, it is useful to place ads to websites which appear in the organic links, as the latter drive even more people to click on the ads.

### Optimizing the distribution of campaign funding

In the section “Priming effect” we demonstrated the priming effect of advertising on terms perceived as less-relevant to vaccines, eliciting future searches for the vaccine. However, our results in the section “Congruence effect of ads” showed that placing an advertisement when a link to the advertiser exists in the organic link dramatically increases the likelihood of a click on the ads.

Some people who already know about the vaccine or even plan to vaccinate, that is, people already deep into the conversation funnel, will be more likely to actually do so if their searches are enhanced through ads placed as a response to their searches on these topics. But, our results suggest that if the goal is to recruit more people to vaccinate, advertisers could prime people through advertising to the less-relevant terms, hitting people in the target population but unaware of the vaccine, not even in the conversion funnel.

To find the division of advertising budgets between searches for the vaccine and less-relevant searches so as to maximize the number of people who vaccinate we consider these two factors as follows: First, the increase in click probability due to priming and second, the increase in click probability due to ads placed for vaccine searches. Specifically, let ${P}_{c}^{v}$ be the probability of click on an ad or organic search result related to vaccination when the user queried for the vaccine, given that a vaccine ad was shown. Similarly, ${P}_{c}^{nv}$ denotes the same, given that the vaccine ad was not shown. For the priming effect, let ${P}_{p}^{v}$ be the probability of click on our ad or a future search for a vaccine, where the search term was not vaccine-related and our ad was shown. ${P}_{p}^{nv}$ denotes the same, but when our ad was not shown.

Increased click probability for vaccine searches is calculated as ${E}_{C}={P}_{c}^{v}-{P}_{c}^{nv}$ and the priming effect, *E*_*P*_, is equal to ${P}_{p}^{v}-{P}_{p}^{nv}$.

The probabilities are shown in Table 6. Note that for influenza, showing the vaccine ads seems to reduce the likelihood of clicks on the ads, but this is likely due to the fact that many people search for the condition without explicitly mentioning the vaccine.

**Table 6:** Change in click probability and future search probability.

Attribute	${P}_{c}^{v}$	${P}_{c}^{nv}$	*E*_*C*_	${P}_{p}^{v}$	${P}_{p}^{nv}$	*E*_*P*_
Influenza	0.5223	0.5811	−0.0588	0.0660	0.0350	0.0310
HPV	0.5298	0.4707	0.0591	0.0526	0.0269	0.0257
Herpes Zoster	0.6036	0.5000	0.1031	0.1181	0.0467	0.0714

Our goal is to maximize *E*_*C*_ ⋅ *α* + *E*_*P*_ ⋅ (1 − *α*), where *α* is the fraction of campaign spending on vaccine searches (*α* ∈ [0, 1]), which is complicated due to the varying prices for any set of keywords. If all keywords were the same price, and *E*_*C*_ > *E*_*P*_, the maximum of this function is reached when relevant ads are shown for all searches for the vaccine, and the remaining budget is given to priming searches. Conversely, when *E*_*C*_ < *E*_*P*_, the function is maximized by showing relevant ads to all priming searches, and the remaining budget spent on vaccine searches. But, in reality prices do differ and for priming to be the more cost-effective option, the price of the cheapest set of priming keywords (where there is an expansive set of possible keywords with diverse pricing) just needs to have a higher expected return per dollar than the cheapest set of keywords for illness or vaccine-related options (which is much more limited in the scope of options). And, keywords later in a conversation funnel, branded keywords in particular, are more generally expensive^29^.

## Discussion

Nudging people towards vaccination is an important public health goal which has received significant attention over the years. Here we focused on methods for finding people to whom vaccine ads should be shown, identifying two main effects of such ads. First, we demonstrated a priming effect, whereby people who might not have otherwise considered vaccination could be nudged towards searching for information on the vaccine by showing vaccination-promoting ads to seemingly irrelevant terms, albeit ones used by the target population. Second, we found a congruence effect, whereby people who had shown an interest in the disease or the vaccine against it were more likely to click on relevant ads or increase the number of future searches following an ad displayed to them. We also found that the vast majority of ads promoting vaccines were targeted at this population.

Finally, we found that there is a complex interplay between organic (unpaid) links and ads. More ads pointing to the same website are correlated with an increasing likelihood of clicks on organic links. Additional organic links from the same website are associated with more clicks on ads in the case of HPV, and less on the organic links in the case of influenza and HPV, but not Herpes Zoster. Similar mixed effects were found in previous work^30–32^, but ours is the first to show that even among different vaccines this effect exists. Thus, advertisers should be careful to take this effect into consideration when designing campaigns.

Our work has several limitations: First, as noted in the Methods, here we chose to focus on two measures of outcome: clicks on relevant ads and future searches for the diseases or the vaccines against them. Though actual vaccination rates would have been a superior outcome, these are difficult (if not impossible) to obtain in our anonymous cohort. However, as shown in Supplementary Data 3, there is a good correlation between vaccination rates and the percentage of people searching for vaccines, when measured at the state level.

Second, our campaigns were run to all users who queried with a relevant keyword. In many cases, it may be possible to focus an advertising campaign by showing it to only the relevant demographics. However, in this paper, we did not test this option as it is unclear to which extent such demographic targeting is accurate and whether it would skew our results.

Third, when asked to quantify term similarity, most people gave scores that were either high (4 and higher) or very low (1–1.5). This was likely due to the difficulty that people have in assigning the middle values to the similarity ranges. Thus, the reported correlations could be skewed because of this difficulty.

Another limitation of our work is that we did not examine the specific attributes of the ads themselves. As noted in the Methods, the ads were chosen from popular ads shown on Microsoft Advertising. However, past work (e.g.,^33,34^), attributes of ads, including for example, whether they contain informational content, appeal to emotion, and include a call to action, all affect how people respond to ads. Another aspect which was not examined in this work was the effect of images which can be included in some forms of advertising, and are known to be useful for eliciting action^35^. These aspects are left for future work.

Research has found that people do not receive recommended vaccinations for a variety of reasons. Mulet Pons et al.^36^ found that people did vaccinate because they didn’t think they needed the vaccine, were unaware of it, were worried by adverse reactions or had just forgotten. Similar results were found by Bricout et al.^37^ for the case of the herpes zoster vaccine. More recently, vaccine hesitancy^38^ was shown to be an important factor. As we show in Supplementary Data 2, a significant percentage of the population are unaware of either the diseases or the vaccines that we studied, even in the target populations. This is in agreement with prior work^39,40^, which found a similar lack of knowledge on these issues. However, our experiments only attempt to alleviate people being unaware of the vaccine (see also Supplementary Data 2). Future research will endeavor to address the other factors cited above through appropriate wording of the ads.

Taken together, our finding have clear implications for the way in which the importance of vaccinations is communicated: If the goal of a campaign is to generate revenue for a particular vaccine manufacturer or provider, it may make sense to focus on the congruence effect, maximize spending on searches near the end of the conversion funnel, that is, on those already searching for the illness or vaccine itself. But, if the goal of a campaign is maximize the number of people in society who receive the vaccine, regardless of type or provider, there is a potentially higher return in focusing on the congruence effect for people in a target population for the vaccine who were searching for something unrelated to the vaccine, but are not already in the conversation funnel. However, as we show (“Optimizing the distribution of campaign funding”), care must be taken to balance the advertising budget across the different parts of the conversion funnel: While there are more people in earlier stages of the funnel, they are also less likely to change their behavior^41^.

This result has implications for digital campaign targeting (especially if future research will show direct measures of vaccine uptake): Digital advertising campaigns should consider not just pushing direct keywords or derive sets of individuals that look exactly like existing customers, but consider tangential keywords that draw in the wider target population who are likely earlier in their conversion funnel, or maybe not even in it. Moreover, we posit that part of the reason for advertiser’s focus on the population which already expressed an intention to vaccinate is that the most common measure for campaign success is click-through rate, which is naturally higher in this population. Therefore, those platforms that run digital advertisements should consider the return on investment measures like the one we note here on expected lift per dollar which is easy enough to automate even for smaller campaigns.

## Methods

In this work, we advertised vaccines against three diseases: Influenza, HPV, and Herpes Zoster. All three vaccines are regularly advertised by pharmaceuticals and health authorities in the USA. The budget for all campaigns was set at a maximum of US$15 per day. Ads were shown to users in the USA.

### Advertising campaign development

Search advertising campaigns comprise of three main elements: Keywords (which searches direct someone to an ad), ad text (words of the ad itself), and landing page (where the ad sends someone that clicks on it).

Ads were shown in response to user queries on Bing which included a campaign keyword, if the bid engine of Microsoft Advertising decided to show said ads. The ads comprise of a short (one sentence) title and a 1–3 sentence body. The ad also includes a link to a website landing page which is displayed to users who click on the advertisement. Ads are shown in one of two locations on the search results page: Top or bottom. They are marked as ads to indicate to users that these are paid search results. We note that search pages also display unpaid links to websites. These are known as organic links.

Each display of an advertisement to a user is referred to as an impression. Advertisers pay whenever an ad is clicked. A common measure of ad performance is the percentage of ads that are clicked by users, also known as the Clickthrough Rate (CTR).

Treatment users in this study were people who queried using the campaign ad keywords and were shown the campaign advertisements. Control users in this study were people who queried using the campaign ad keywords but were not shown the campaign advertisements. Control users saw either no ads or ads from other advertisers, as served by the advertising system. The bids were set by the advertisement system to be the smallest values that would still make the ads appear on the first page of the search results, making the assignment to the treatment and control semi-random. As no demographic information is available on the populations, we cannot verify that the randomization was completely effective.

Below we provide details on how each element of the campaigns was selected for each of the vaccines.

Keywords for the influenza vaccine comprised of three types: Flu vaccine keywords, including mentions of the flu vaccine or the just the flu, and seasonal keywords. All three campaigns have a similar pattern of vaccine and illness-related terms, targeting people who are inside the conversation funnel, and some set of tangentially related terms (which differ slightly in structure to match the illness), targeting people who are in the target population, but may be outside the conversion funnel. In this case the latter was found by selecting searches with the following keywords that had at least 1000 daily searches in October/November 2018 and had similar levels of search across both 2017 and 2018: “weather”, “fall”, “autumn”, “pumpkin”, “halloween”, “thanksgiving”, “turkey”, “gravy”, “daylight saving”.

The text of the ads was developed by the authors, after examining other influenza vaccine ads shown on Microsoft Advertising. All ads led to the HealthMap Vaccine Finder website https://vaccinefinder.org/.

Keywords for the HPV vaccine were derived from the following classes of terms:

- Vaccine-related: Commercial names of the HPV vaccine or the term “hpv vaccine”
- Disease-related: HPV, “human papilloma virus”
- Keywords of other HPV vaccine ads: Other keywords used by advertisers of the HPV vaccine on Microsoft Advertising, e.g., “cervical cancer vaccine” and “signs of cervical cancer”
- High-school related: Keywords related to college applications (e.g., “common app”, “gpa”, “scholarship”), standardized testing (“sat”,“ap”), parent-school district connection software (“parent portal”, “infinite campus”)
- Parent queries: We identified people who were likely parents by finding those people who queried on Bing between 1 October 2018 and 31 March 2019, and mentioned the terms “my teen”, “teenage son”, or “teenage daughter”. We then scored queries during that time range as the fraction of users who made each query and were in the parent population. The top 50 queries were included as keywords.
- Weather- and news-related terms, as in the influenza vaccine adverts.

Advertisements comprised of the text of the most popular HPV vaccine ads shown on Microsoft Advertising on September 2018. The landing page for the ads was the Center for Disease Control (CDC) page on the HPV vaccine, https://www.cdc.gov/HPV/parents/vaccine.html.

Keywords for the Herpes Zoster vaccine were derived from the following classes of terms:

- Vaccine-related: Commercial names of the Herpes Zoster vaccine or the term “zoster vaccine”.
- Disease-related: “shingles”, “herpes zoster”.
- Older people’s queries: We found the 50 queries made by the highest percentage of people aged 50 or older during October 2019. Age was provided by users at the time of registration to Bing.
- Weather- and news-related terms, as in the influenza vaccine adverts.

Advertisements comprised of the text of the most popular Herpes Zoster vaccine ads shown on Microsoft Advertising on September 2018. The landing page for the ads was the CDC page on the Herpes Zoster vaccine, https://www.cdc.gov/shingles/vaccination.html.

### Assessing campaign effectiveness

As noted in the Introduction, directly measuring vaccination rates as a result of advertising is difficult. Therefore, here we use two other proxies for behavior change: Clicks on the ads (as measured by the CTR) and future searches by the same user on a search engine. The latter measure was the percentage of users who queried for the vaccine on Bing, either by name or by general terms, e.g., “flu vaccine”, following the display of a relevant ad. The latter is a common proxy for medically related behavior change^6–8^. As shown in Supplementary Data 3, at a state-level, this latter measure is a reasonable proxy for vaccination rates.

Searches of users in this experiment were anonymized before the investigators had access to them. Each search comprised of the time and date of the search, an anonymous user identifier, and the query text. We define condition searches as those searches that contained keywords related to either the vaccine or the disease. Data were extracted for the duration of the advertising campaigns.

### Classification of search term similarity

We used Amazon Mechanical Turk to code the search terms on a 5-point Likert scale to classify if the intent of the search was aimed towards a vaccine or not. The scale ran from “Definitely NOT searching for [vaccine]” to “Definitely searching for [vaccine]”. For background we provided the first paragraph of the Wikipedia article on the vaccine and encourage the workers to search if they need more information. For each vaccine we coded the term with over 100 ad impressions or (in the case of HPV) the top 25 terms by impression. We asked six workers to classify each term and took the average score given by them.

### Trial registration and IRB approval

This study was approved by the Microsoft Institutional Review Board. Informed consent could not be received from the anonymous participants. This trial was registered on AsPredicted registration number 34050.

### Reporting summary

Further information on research design is available in the Nature Research Reporting Summary linked to this article.

## Supplementary information

Supplementary Information

Reporting Summary

## Data Availability

The data that support the findings of this study are available from Microsoft, but restrictions apply to the availability of the data. Specifically, all aggregate advertising data are available from the authors on reasonable request. Individual-level search data are available from the authors on reasonable request and with the permission of Microsoft.

## Code availability

Code is available from the authors on reasonable request.

## References

[1] Organization, W. H. Immunization coverage. https://www.who.int/en/news-room/fact-sheets/detail/immunization-coverage (2019).

[2] Centers for Disease Control and Prevention. Vaccination coverage among adults in the united states, national health interview survey, 2017. https://www.cdc.gov/vaccines/imz-managers/coverage/adultvaxview/pubs-resources/NHIS-2017.html (2017).

[3] Garcia-Molina, H., Koutrika, G. & Parameswaran, A. Information seeking: Convergence of search, recommendations and advertising. *Communications of the ACM* (2011).

[4] Statista: Global no.1 business data platform. https://www.statista.com/outlook/219/109/search-advertising/united-states#market-revenue (2019).

[5] Statista: Global no.1 business data platform. https://www.statista.com/outlook/219/100/search-advertising/worldwide#market-revenue (2019).

[6] Yom-Tov E, Muennig P, El-Sayed AM (2016). Web-based antismoking advertising to promote smoking cessation: A randomized controlled trial. J. Med. Internet Res..

[7] Yom-Tov E, Shembekar J, Barclay S, Muennig P (2018). The effectiveness of public health advertisements to promote health: A randomized-controlled trial on 794,000 participants. NPJ Digital Med..

[8] Yom-Tov E, Brunstein-Klomek A, Mandel O, Hadas A, Fennig S (2018). Inducing behavioral change in seekers of pro-anorexia content using internet advertisements: Randomized controlled trial. JMIR Mental Health.

[9] Lewis RA, Reiley DH (2014). Online ads and offline sales: measuring the effect of retail advertising via a controlled experiment on Yahoo! Quantitative Market. Economics.

[10] Simmering JE, Polgreen LA, Polgreen PM (2014). Web search query volume as a measure of pharmaceutical utilization and changes in prescribing patterns. Res. Social Administrative Pharmacy.

[11] Ayers JW, Ribisl KM, Brownstein JS (2011). Tracking the rise in popularity of electronic nicotine delivery systems (electronic cigarettes) using search query surveillance. Am. J. Preventive Med..

[12] Liu T, He G, Lau A (2018). Avoidance behavior against air pollution: evidence from online search indices for anti-pm 2.5 masks and air filters in Chinese cities. Environ. Economic Policy Studies.

[13] Althouse BM, Ng YY, Cummings DA (2011). Prediction of dengue incidence using search query surveillance. PLoS Negl Trop Dis..

[14] Lampos, V. et al. Tracking covid-19 using online search. *NPJ Digital Medicine*. 10.1038/s41746-021-00384-w (2020). 10.1038/s41746-021-00384-w PMC7870878 33558607

[15] Sarigul, S. & Rui, H. Nowcasting obesity in the us using Google search volume data. *Tech. Rep*. (2014).

[16] Ofran Y, Paltiel O, Pelleg D, Rowe JM, Yom-Tov E (2012). Patterns of information-seeking for cancer on the internet: An analysis of real-world data. PLoS ONE.

[17] Choi H, Varian H (2012). Predicting the present with Google trends. Economic Record.

[18] Kim S (2016). Forecasting short-term air passenger demand using big data from search engine queries. Automation Construction.

[19] Ettredge M, Gerdes J, Karuga G (2005). Using web-based search data to predict macroeconomic statistics. Commun. ACM.

[20] Street A, Murray TA, Blitzer J, Patel RS (2015). Estimating voter registration deadline effects with web search data. Political Analysis.

[21] Kristoufek L, Moat HS, Preis T (2016). Estimating suicide occurrence statistics using Google trends. EPJ Data Sci..

[22] Mohanty S, Leader AE, Gibeau E, Johnson C (2018). Using Facebook to reach adolescents for human papillomavirus (HPV) vaccination. Vaccine.

[23] Jamison AM (2020). Vaccine-related advertising in the Facebook ad archive. Vaccine.

[24] Jerath K, Ma L, Park Y-H (2014). Consumer click behavior at a search engine: the role of keyword popularity. J. Marketing Res..

[25] Joo M, Wilbur KC, Zhu Y (2016). Effects of TV advertising on keyword search. Int. J. Res. Marketing.

[26] Lu X, Zhao X (2014). Differential effects of keyword selection in search engine advertising on direct and indirect sales. J. Management Inf. Syst..

[27] Naik PA, Mantrala MK, Sawyer AG (1998). Planning media schedules in the presence of dynamic advertising quality. Marketing Sci..

[28] Keane MT, O’Brien M, Smyth B (2008). Are people biased in their use of search engines?. Commun. ACM.

[29] Blake T, Nosko C, Tadelis S (2015). Consumer heterogeneity and paid search effectiveness: A large-scale field experiment. Econometrica.

[30] Yang S, Ghose A (2010). Analyzing the relationship between organic and sponsored search advertising: Positive, negative, or zero interdependence?. Marketing Sci..

[31] Chan, D., Kumar, D., Ma, S. & Koehler, J. Impact of ranking of organic search results on the incrementality of search ads. http://static.googleusercontent.com/media/research.google.com/en//pubs/archive/37731.pdf (2012).

[32] Danescu-Niculescu-Mizil, C., Broder, A. Z., Gabrilovich, E., Josifovski, V. & Pang, B. Competing for users’ attention: On the interplay between organic and sponsored search results. In *Proc. 19th Int. Conference on World Wide Web*, 291–300 (2010).

[33] Chandrasekaran D, Srinivasan R, Sihi D (2018). Effects of offline ad content on online brand search: Insights from super bowl advertising. J. Acad. Marketing Sci..

[34] Youngmann, B., Yom-Tov, E., Gilad-Bachrach, R. & Karmon, D. The automated copywriter: Algorithmic rephrasing of health-related advertisements to improve their performance. In *Proc. The Web Conference 2020*, 1366–1377 (2020).

[35] Huhmann BA, Franke GR, Mothersbaugh DL (2012). Print advertising: executional factors and the RPB grid. J. Business Res..

[36] MJ MP (1995). Evaluation of the completion of influenza vaccination. Atencion Primaria.

[37] Bricout H (2019). Determinants of shingles vaccine acceptance in the United Kingdom. PLoS ONE.

[38] Dubé E (2013). Vaccine hesitancy: An overview. Hum Vaccin Immunother..

[39] McBride KR, Singh S (2018). Predictors of adults’ knowledge and awareness of HPV, HPV-associated cancers, and the HPV vaccine: Implications for health education. Health Educ. Behav..

[40] Lu P-J (2017). Awareness among adults of vaccine-preventable diseases and recommended vaccinations, United States, 2015. Vaccine.

[41] Hoban PR, Bucklin RE (2015). Effects of internet display advertising in the purchase funnel: Model-based insights from a randomized field experiment. J. Marketing Res.

