# Supplementary material for: Vaccine advertising: preach to the converted or to the unaware?
Source: NPJ Digit Med. 2021 Feb 11;4:23. doi: 10.1038/s41746-021-00395-7 (PMC7878497; doi:10.1038/s41746-021-00395-7)
Supplement: Supplementary file 1 — Supplementary Information [file 41746_2021_395_MOESM1_ESM.pdf]

Supplementary Information for Vaccine advertising: Preach to the converted or to the unaware?

Masha Krupenkin[*], Elad Yom-Tov[†], David Rothschild[‡]

December 22, 2020

Overview

Supplementary Data 1 provides a listing of keywords and their similarity ratings, as provided by the crowdsourced workers.

Supplementary Data 2 provides evidence that many people in the target populations are unaware of vaccines that they should receive. Supplementary Data 3 provides supporting evidence for the claim that searches for the vaccine are indicative of an intention to vaccinate.

Finally, Supplementary Data 4 details another test that we conducted, to see if indications of having influenza in the previous season could be correlated with search behavior related to flu vaccines.

Supplementary Data 1: Sample of the coded campaign keywords for their relevance to the disease or vaccine

Figure 1 shows the distribution of term similarity scores for the keywords of the three campaigns. Table 1 shows the five most similar and five most dissimilar terms, as rated by crowdsourced workers, for each vaccine.

[*]Boston College, Boston MA, USA
[†]Microsoft Research, Herzeliya, Israel. email: eladyt@microsoft.com
[‡]Microsoft Research, New York NY, USA.

Vaccination	Term	Term similarity
Influenza	flu vaccine	4.8
	flu shot side effects	4.5
	flu shot	4.4
	cdc flu vaccine	4.2
	flu	4.2
	pumpkin	1.0
	halloween haunted houses	1.0
	turkey stuffing	1.0
	cooking turkey	1.0
	wunderground weather	1.0
HPV	human papilloma virus	5.0
	guardasil	5.0
	guardacil	5.0
	cervical cancer vaccine	5.0
	vph vaccine	4.7
	common app	1.0
	khan academy sat	1.0
	accuweather	1.0
	weather	1.0
	weather underground	1.0
Herpes Zoster	zoster vaccine	3.8
	shingles	3.3
	shingrix	3.2
	www.msn.com	2.3
	schwab	2.0
	weather	1.0
	weather forecast	1.0
	wish	1.0
	www.aol.com	1.0
	www.fidelity.com	1.0

Table 1: Five most similar and most dissimilar terms per vaccination, as rated by crowdsourced workers. Similarity scores are averages for the scores given by the workers.

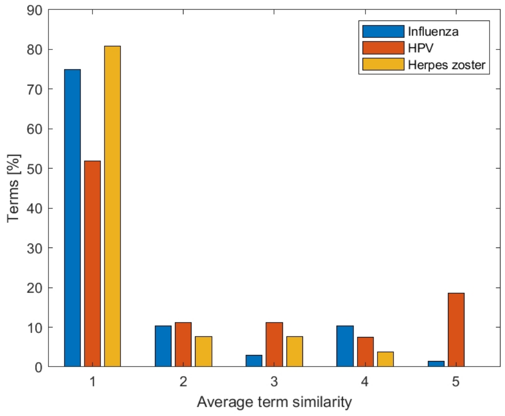

Figure 1: Distribution of term similarity scores for keywords of the three campaigns

Supplementary Data 2: Awareness of the studied conditions and the vaccines against them

We ran a poll of a randomized sample of Americans 18 years or older, weighted to a standard demographic battery, about knowledge of the three diseases and vaccines. We also asked about 5 control diseases (measles, chicken pox, HIV/AIDS, Lyme disease and COVID-19, for three of which there are no (currently) available vaccines (HIV/AIDS, Lyme disease and COVID-19).

The respondents were recruited through Pollfish, which administers surveys in native ad units of mobile and desktop applications. We surveyed 500 respondents total. To avoid any confusion the survey provided both the formal and colloquial name for of the each of the diseases tested.

Figure 2 shows the percentage of people who responded that they know of each of the diseases and (separately) that they are aware of a vaccine for said disease, as a function of age.

Focusing in on the most impacted populations, 96% of people 55 or older know of herpes zoster and of influenza, but only 73% know herpes zoster has a vaccine and 79% that influenza does (younger people are way less knowledgeable on both, even though flu can affect all ages). Knowledge of the HPV vaccine is just 37% among 18-24 year-olds and 57% among 25-34 year-olds, where 72% and 65% respectively know of HPV. Under 20% thought there is a vaccine for the 3 control condition which, while encouraging, is not much lower than, for example, the percentage of younger people who knew about the herpes zoster vaccine. We note that these results are in agreement with prior work [1, 2], which found similar lack of knowledge on these vaccines.

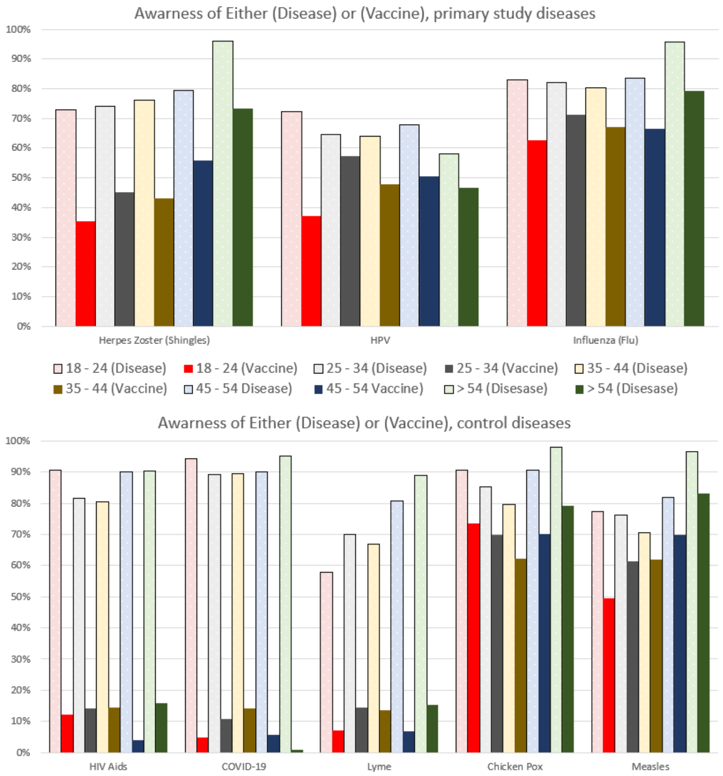

Figure 2: Percentage of people at each age group who responded that they are aware of the disease (lighter shades) and of a vaccine for the disease (darker shades). At the time of writing, HIV/AIDS, lyme disease and COVID-19 do not have a vaccine.

Vaccination	All queries	Personal references
Influenza	0.54	0.40
HPV	0.34	0.14
Herpes Zoster	0.64	0.57

Table 2: Correlation between state-level vaccination rates and the percentage of Bing users searching for the vaccine at each state.

Supplementary Data 3: Correlation between searches for vaccines and vaccination rates

We evaluated the correlation between queries for vaccines and the vaccination rates at the state level in the US. State-level vaccination rates were derived from the following CDC sources:

- Influenza: 2018-19 Influenza Season Vaccination Coverage Report for December 2018 [3].
- HPV: 2018 Adolescent Human Papillomavirus (HPV) Vaccination Coverage Report [4].
- Herpes Zoster: 2008 through 2017 Adult Vaccination Coverage Trend Report [5].

These were compared to the percentage of people from each state which used to query Bing between September 1st and December 31st, 2018 for the following:

- Influenza: ”flu” and ”vaccine”
- HPV: Either ”HPV” (and common spelling variations thereof) or ”cervical cancer” and ”vaccine” or the vaccine brand names (Guardasil, Cervarix)
- Herpes Zoster: Either ”shingles” or ”zoster” and ”vaccine” or the vaccine brand names (zvl, shingrix, zostavax).

The correlation between the percentage of people querying and the state-level vaccination rates is shown in Table 2, stratified by all queries and by queries which also included a personal reference (”I”, ”me”, or ”my”). As the table shows, there is high correlation between queries for vaccination and vaccination rates, suggesting that queries for vaccine could be a useful proxy for people’s intention to vaccinate. We note that in this analysis we did not consider factors known to affect vaccination rates [6, 7] such as income, education, access to healthcare, race, and other factors which may affect vaccination levels and could have improved the observed correlation, as our focus is the correlation of search queries and vaccination rates.

Supplementary Data 4: Model of influenza with previous year

We attempted to estimate the effect of influenza-like illness (ILI) in a previous season on the ability to elicit clicks on vaccine-related ads for a flu vaccine. To do so, we first identified users who likely experienced ILI in a previous season and used this information as an independent variable to a model of clicks and (separately) future vaccine queries.

We followed the procedure described in Yom-Tov et al. [8] to identify people who likely experienced ILI. Specifically, we extracted the percentage of users at each state who queried Bing each week for each of the terms described below from September 1st, 2017 to 16 September 2019. The terms were 217 flu-related terms listed in Lampos et al. [9].

We trained a lasso regression model [10] to predict the CDC's U.S. Outpatient Influenza-like Illness Surveillance Network (ILINet) rate of ILI for the same dates as the query data. We used the data up to December 31st, 2018 as training data and the remaining for test the model's performance.

The model achieved an R^2 of 0.35. The 10 terms with the highest weight were (in descending order of weight) "banging headache", "coughed", "biggest headache", "dying flu", "gonna vomit", "nose sore throat", "sneezed", "bad cough", "runny nose", and "chesty cough".

We identified people as likely experiencing ILI in a previous season as those people who queried at least once about any of the above-mentioned 10 terms between September 1st 2018 and April 30th, 2019. Similarly, people who queried for the flu vaccine during that time were marked as having expressed interest in the flu vaccine in the previous season. We note that in addition to identification using the most important model keywords, an additional classifier trained to identify personal statements of illness was employed by Yom-Tov et al. [8]. The short length of search queries often makes it impossible to identify such personal statements. For this reason, we did not use this additional stage here, possibly adding noise to the data.

Among users in the study, 21,310 asked about the vaccine in the previous season and 1,800,573 did not. Additionally, 6439 users mentioned one of the 10 ILI terms and 1,815,444 did not.

Table 3 shows the parameters of a logistic regression model for clicks and of a Cox proportional hazard model for future vaccine searches for those users who were active during the 2018-2019 flu season. As the models show, both queries for a vaccine in the previous season and ILI queries during the same reduced the likelihood of both clicks and future searches.

References

[1] McBride, K. R. & Singh, S. Predictors of adults' knowledge and awareness of hpv, hpv-associated cancers, and the hpv vaccine: implications for health education. *Health Education & Behavior* **45**, 68–76 (2018).

Variable	Click (Odds ratio)	Future search (Hazard ratio)
1. Queried for vaccines last year	0.72	0.75
2. ILI last year	0.82	0.79
3. Interaction of (1) and (2)	0.84	1.04^{NS}
4. Disease query	0.99^{NS}	2.70
5. Disease ad	0.98	1.16
6. Vaccine query	1.02	2.43
7. Vaccine ad	1.45	0.95
8. Campaign ad	1.02^{NS}	1.30
9. Ad rank	1.01	1.01
10. Previous ads to this user	1.00	1.00^{NS}

Table 3: Logistic regression model parameters for clicks (left) and a Cox proportional hazard model parameters for future vaccine searches (right). All parameters are statistically significant ($P < 0.05$, except values denoted by N.S.

[2] Lu, P.-j. *et al.* Awareness among adults of vaccine-preventable diseases and recommended vaccinations, united states, 2015. *Vaccine* **35**, 3104–3115 (2017).

[3] for Disease Control, C. & Prevention. Influenza season vaccination coverage report. `https://www.cdc.gov/flu/fluvaxview/reportshtml/reporti1819/reporti/index.html` (2019). Accessed on July 2019.

[4] for Disease Control, C. & Prevention. Human papillomavirus (hpv) vaccination coverage among adolescents 13-17 years by state, hhs region, and the united states, national immunization survey-teen (nisteen), 2018. `https://www.cdc.gov/vaccines/imz-managers/coverage/teenvaxview/data-reports/hpv/reports/2018.html` (2018). Accessed on July 2019.

[5] for Disease Control, C. & Prevention. Pneumococcal vaccination coverage among adults 18–64 years at increased risk and 65 years, td and tdap vaccination coverage among adults 18 years, and shingles vaccination coverage among adults 60 years by selected local area, state, hhs region, and the united states, brfss, 2008 through 2017. `https://www.cdc.gov/vaccines/imz-managers/coverage/adultvaxview/data-reports/general-population/trend/index.html` (2019). Accessed on July 2019.

[6] Lu, P.-j. *et al.* Racial and ethnic disparities in vaccination coverage among adult populations in the us. *Vaccine* **33**, D83–D91 (2015).

[7] Walker, T. Y. *et al.* National, regional, state, and selected local area vaccination coverage among adolescents aged 13–17 years—united states, 2017. *Morbidity and Mortality Weekly Report* **67**, 909 (2018).

[8] Yom-Tov, E., Johansson-Cox, I., Lampos, V. & Hayward, A. C. Estimating the secondary attack rate and serial interval of influenza-like illnesses using social media. *Influenza and other respiratory viruses* **9**, 191–199 (2015).

[9] Lampos, V., Yom-Tov, E., Pebody, R. & Cox, I. J. Assessing the impact of a health intervention via user-generated internet content. *Data Mining and Knowledge Discovery* **29**, 1434–1457 (2015).

[10] Tibshirani, R. Regression shrinkage and selection via the lasso. *Journal of the Royal Statistical Society: Series B (Methodological)* **58**, 267–288 (1996).
