# Supplementary material for: Vaccine advertising: preach to the converted or to the unaware?
Source: NPJ Digit Med. 2021 Feb 11;4:23. doi: 10.1038/s41746-021-00395-7 (PMC7878497; doi:10.1038/s41746-021-00395-7)
Supplement: Supplementary file 2 — Reporting Summary [file 41746_2021_395_MOESM2_ESM.pdf]

nature research

Corresponding author(s): Elad Yom-Tov

Last updated by author(s): Nov 10, 2020

Reporting Summary

Nature Research wishes to improve the reproducibility of the work that we publish. This form provides structure for consistency and transparency in reporting. For further information on Nature Research policies, see our Editorial Policies and the Editorial Policy Checklist.

Statistics

For all statistical analyses, confirm that the following items are present in the figure legend, table legend, main text, or Methods section.

n/a	Confirmed	
☐	☒	The exact sample size (n) for each experimental group/condition, given as a discrete number and unit of measurement
☐	☒	A statement on whether measurements were taken from distinct samples or whether the same sample was measured repeatedly
☐	☒	The statistical test(s) used AND whether they are one- or two-sided<br>*Only common tests should be described solely by name; describe more complex techniques in the Methods section.*
☐	☒	A description of all covariates tested
☐	☒	A description of any assumptions or corrections, such as tests of normality and adjustment for multiple comparisons
☒	☐	A full description of the statistical parameters including central tendency (e.g. means) or other basic estimates (e.g. regression coefficient) AND variation (e.g. standard deviation) or associated estimates of uncertainty (e.g. confidence intervals)
☐	☒	For null hypothesis testing, the test statistic (e.g. F, t, r) with confidence intervals, effect sizes, degrees of freedom and P value noted<br>*Give P values as exact values whenever suitable.*
☒	☐	For Bayesian analysis, information on the choice of priors and Markov chain Monte Carlo settings
☒	☐	For hierarchical and complex designs, identification of the appropriate level for tests and full reporting of outcomes
☐	☒	Estimates of effect sizes (e.g. Cohen's d, Pearson's r), indicating how they were calculated

Our web collection on statistics for biologists contains articles on many of the points above.

Software and code

Policy information about availability of computer code

Data collection	Data was collected through Microsoft's internal systems.
Data analysis	Matlab 2020 and Python 3.7

For manuscripts utilizing custom algorithms or software that are central to the research but not yet described in published literature, software must be made available to editors and reviewers. We strongly encourage code deposition in a community repository (e.g. GitHub). See the Nature Research guidelines for submitting code & software for further information.

Data

Policy information about availability of data

All manuscripts must include a data availability statement. This statement should provide the following information, where applicable:

- Accession codes, unique identifiers, or web links for publicly available datasets
- A list of figures that have associated raw data
- A description of any restrictions on data availability

The data that support the findings of this study are available from Microsoft, but restrictions apply to the availability of the data. Specifically, all aggregate advertising data are available from the authors on reasonable request. Individual-level search data are available from the authors on reasonable request and with permission of Microsoft.

Field-specific reporting

Please select the one below that is the best fit for your research. If you are not sure, read the appropriate sections before making your selection.

☐ Life sciences ☒ Behavioural & social sciences ☐ Ecological, evolutionary & environmental sciences

For a reference copy of the document with all sections, see nature.com/documents/nr-reporting-summary-flat.pdf

Behavioural & social sciences study design

All studies must disclose on these points even when the disclosure is negative.

Study description	We conducted a pre-registered online ads campaign designed to encourage people to vaccinate against 3 diseases: influenza (flu), human papillomavirus (HPV), and herpes zoster (shingles). Analysis is quantitive.
Research sample	The ads were shown to approximately 69 thousand people and were compared to similar ads shown to 8.6 million people, all in the USA. The sample is representative of internet users who searched for the relevant terms.
Sampling strategy	Ads were run for the time specified in the paper. All users who were exposed to the ads or to similar ads were analysed in this work.
Data collection	Data collection was through Microsoft's systems.
Timing	Listed in Table 1 of the paper.
Data exclusions	None
Non-participation	None
Randomization	Randomization was based on the advertising system choosing to show the ads. Investigators had no control over this syste,.

Reporting for specific materials, systems and methods

We require information from authors about some types of materials, experimental systems and methods used in many studies. Here, indicate whether each material, system or method listed is relevant to your study. If you are not sure if a list item applies to your research, read the appropriate section before selecting a response.

Materials & experimental systems

n/a	Involved in the study	
☒	☐	Antibodies
☒	☐	Eukaryotic cell lines
☒	☐	Palaeontology and archaeology
☒	☐	Animals and other organisms
☐	☒	Human research participants
☒	☐	Clinical data
☒	☐	Dual use research of concern

Methods

n/a	Involved in the study	
☒	☐	ChIP-seq
☒	☐	Flow cytometry
☒	☐	MRI-based neuroimaging

Human research participants

Policy information about studies involving human research participants

Population characteristics	Internet users in the US.
Recruitment	Recruitment was based on the use of specific terms in search queries, as listed in the paper.
Ethics oversight	This study was approved by the Microsoft Institutional Review Board

Note that full information on the approval of the study protocol must also be provided in the manuscript.
